# Editorial: Plant molecular farming for the production of next-generation vaccines and biologics – prospects and challenges

**DOI:** 10.3389/fpls.2024.1381234

**Published:** 2024-02-27

**Authors:** Balamurugan Shanmugaraj, Kathleen Hefferon

**Affiliations:** ^1^ Department of Pharmacognosy and Pharmaceutical Botany, Faculty of Pharmaceutical Sciences, Chulalongkorn University, Bangkok, Thailand; ^2^ Department of Microbiology, Cornell University, Ithaca, NY, United States

**Keywords:** plant molecular farming, vaccines, nicotiana, biologics, plants

Plant molecular farming, meaning the use of plants as production platforms for the generation of vaccines, monoclonal antibodies and other therapeutic agents, continues to expand and mature (Shanmugaraj et al., 2020). This multidisciplinary field draws knowledge from the researchers with expertise in plant science, medicine and the pharmaceutical sciences, and regularly engages with the academic, government and industrial sectors. Plants have been cultivated as sources of medicine for ages, and novel approaches based on synthetic biology have advanced the use of plants to generate biologics of all sorts, thus wedding modern medicine to its historical past. Vaccines produced in plants are efficacious, inexpensive and incorporates post translational modifications not found in bacterial systems, yet lack the risk of human pathogen contamination, unlike their mammalian production system counterparts. Plant systems can easily be scaled up, can be stored at ambient temperatures and their facile use makes them excellent candidates for low to middle income countries (He et al., 2021; Lobato Gómez et al., 2021).

This Research Topic covers a variety of subjects that are highly relevant for plant made pharmaceuticals at their current stage of development. One common theme that comes through is the production of monoclonal antibodies for a variety of medical purposes. For example, in Improving the efficacy of plant-made anti-HIV monoclonal antibodies for clinical use, Grandits et al. describe how *Nicotiana benthamiana* plants were utilized to produce three broadly neutralizing antibodies (mAbs which block virus attachment and entry) as an alternative to or combination with antiretroviral therapy. The authors engineered plant-made bNAbs which were greater in efficacy and exhibited an improved pharmacokinetic profile. Cocktails of these antibodies could mitigate the high cost and availability issues of anti-HIV therapies where HIV-1 is found in greatest abundance, low- to middle- income countries. The manuscript ‘Anti-tumor effect of plant-produced anti-CTLA-4 monoclonal antibody in a murine model of colon cancer,’ by Bulaon et al. describes the production of an anti-CTLA-4 2C8 mAbs to cytotoxic T lymphocyte-associated protein 4 in *Nicotiana benthamiana* plants and examination of their use as a cancer immunotherapy in knocked-in mice with colon tumors. The results of this study indicate that plant-based versions of mAbs could overcome the exorbitant costs associated with cancer care, particularly in developing countries.

Advances in glycan engineering for plant production of biologics also predominate this Research Topic. In Production and N-glycan engineering of Varlilumab in *Nicotiana benthamiana*, by Nguyen et al., an N-glycan-engineered version of the CD27-targeting monoclonal antibody Varlilumab was expressed in *Nicotiana benthamina* plants. Similarly, Beihammer et al. incorporated a protozoan oligosaccharyltransferase to improve its ability to glycosylate the Fc of IgG1.

Contributions by Margolin et al., Mamedov et al. and Ruocco et al. address the SARS-CoV-2 pandemic in multiple ways. Margolin et al. used an integrated host and glyco-engineering approach, NXS/T Generation™, to produce SARS-CoV-2 prefusion spike trimers in *Nicotiana benthamiana*, and compared glycan profile as well as immunogenicity to mammalian counterparts, while Mamedov et al. produced an RBD variants cocktail in Nicotiana plants that worked effectively against both Omicron and Delta variants. Ruocco et al. describe the analysis of six different RBD variants produced in plants in terms of yield and N-glycan profiles.

This Research Topic also addresses other aspects of molecular farming, such as food-borne illness. In Accumulation of colicin M protein and its biological activity in transgenic lettuce and mizuna plants, by Shcherbak et al., antimicrobial protein Colicin M (ColM) was expressed in lettuce and mizuna plants. Extracts from these plants demonstrated effectiveness in blocking infection by two noteworthy strains of EHEC as well as several anti-biotic resistant strains of *E. coli*. A preliminary study of the immunogenic response of plant-derived multi-epitopic peptide vaccine candidate of *Mycoplasma gallisepticum* in chickens, Mugunthan et al. cover the response in poultry to a plant-made vaccine to *Mycoplasma gallisepticum* (MG), and avian respiratory infection. Finally, in Artificial intelligence-driven systems engineering for next-generation plant-derived biopharmaceuticals, Parthiban et al. explore the integration of recombinant protein engineering with AI-based machine learning and deep learning algorithms through the lens of plant made pharmaceutical production. Using this approach, the authors gather insight into protein folding and stability, glycan profiles, protein activity and organelle targeting.

The number of papers published in this Research Topic, coupled with the wide variety of applications described, illustrates the relevance of molecular farming today. As plant -made pharmaceuticals evolve, they will continue to address the many urgent issues that we face in global health for many years to come.

## Author contributions

BS: Writing – original draft, Writing – review & editing. KH: Writing – original draft, Writing – review & editing.
